# Assessing the effects of survey-inherent disturbance on primate detectability: Recommendations for line transect distance sampling

**DOI:** 10.1007/s10329-022-01039-4

**Published:** 2022-12-09

**Authors:** Mattia Bessone, Hjalmar S. Kühl, Gottfried Hohmann, Ilka Herbinger, K. Paul N’Goran, Papy Asanzi, Pedro B. Da Costa, Violette Dérozier, D. B. Ernest Fotsing, B. Beka Ikembelo, D. Mpongo Iyomi, B. Iyomi Iyatshi, Pierre Kafando, A. Mbangi Kambere, B. Dissondet Moundzoho, L. Kako Musubaho, Barbara Fruth

**Affiliations:** 1 School of Biological and Environmental Sciences, Liverpool John Moores University, Liverpool, L3 3AF UK; 2 Faculty of Biology/Department of Neurobiology, Ludwig Maximilian University of Munich, Großhaderner Straße 2, 82152 Planegg-Martinsried, Germany; 3 Department of Ecology of Animal Societies, Max Planck Institute of Animal Behavior, Bücklestraße 5, 78467 Constance, Germany; 4 Department of Primatology, Max Planck Institute for Evolutionary Anthropology, Deutscher Platz 6, 04103 Leipzig, Germany; 5 German Centre for Integrative Biodiversity Research (iDiv) Halle-Leipzig-Jena, 04103 Leipzig, Germany; 6 Legacy Landscapes Fund (LLF), c/o NTA, 60325 Frankfurt am Main, Germany; 7 Department of Africa and South America, WWF Germany, Reinhardtstraße. 18, 10117 Berlin, Germany; 8 WWF Regional Office for Africa–Yaoundé Hub/Cameroon Country Programme Office, PO Box 6776, Yaoundé, Cameroon; 9 Department of Biology, University of Fribourg, Chemin du Musée 10, CH-1700 Fribourg, Switzerland; 10 Primate Expertise No. 011, Av. de La Cathédrale, Quartier Ndendere, Democratic Republic of the Congo; 11 Institut Congolais Pour La Conservation de La Nature (ICCN), 13 Avenue Des Cliniques, Kinshasa, Democratic Republic of the Congo; 12 WWF in the Democratic Republic of the Congo, 14, Avenue Sergent Moke, Commune de Ngaliema, Kinshasa, Democratic Republic of the Congo; 13 Centre for Research and Conservation, Royal Zoological Society of Antwerp, B-2018 Antwerp, Belgium; 14 Biodiversity & Landscape Unit, University of Liège, Gembloux Agro-Bio Tech, 2 Passage des Déportés, 5030 Gembloux, Belgium

**Keywords:** *Cercopithecus*, *Colobus*, *Lophocebus*, Salonga National Park, Density estimates, Disturbance

## Abstract

Habitat destruction and over-hunting are increasingly threatening the arboreal primates of Central Africa. To establish effective conservation strategies, accurate assessments of primate density, abundance, and spatial distribution are required. To date, the method of choice for primate density estimation is line transect distance sampling. However, primates fleeing human observers violate methodological assumptions, biasing the accuracy of resulting estimates. In this study, we used line transect distance sampling to study five primate species along 378 km of transects in Salonga National Park, Democratic Republic of the Congo. We tested the effect of different levels of survey-inherent disturbance (i.e., cutting) on the number of observed (i) primate groups, and (ii) individuals within groups, by counting groups at three different time lags after disturbance of the transect, (i) a minimum of 3 h, (ii) 24 h, (iii) a minimum of 3 days. We found that survey-inherent disturbance led to underestimated densities, affecting both the number of encountered groups and of observed individuals. However, the response varied between species due to species-specific ecological and behavioral features. *Piliocolobus tholloni* and *Colobus angolenis* resumed an unaltered behavior only 24 h after disturbance, while *Lophocebus aterrimus*, *Cercopithecus ascanius*, and *Cercopithecus wolfi* required a minimum of 10 days. To minimize bias in density estimates, future surveys using line transect distance sampling should be designed considering survey-inherent disturbance. We recommend evaluating the factors driving primate response, including habitat type, niche occupation, and hunting pressure, peculiar to the survey-specific area and primate community under study.

**Supplementary Information:**

The online version contains supplementary material available at 10.1007/s10329-022-01039-4.

## Introduction

The effective conservation of wild animal populations requires accurate estimates of their distribution, density, and abundance, information critical to the assessment of population status and temporal trends (Nichols and Williams 2006). Consequently, for obtaining unbiased estimates, reliable field methods are equally crucial to correctly informing conservation strategies.

This is particularly true for taxa of high conservation importance, such as primates (Chapman et al. 2020). There is a rich body of literature describing methods and best practices for great ape density estimates (Kühl et al. 2008), iconic species suffering catastrophic population declines (Carvalho et al. 2021; Junker et al. 2012; Kühl et al. 2017; Plumptre et al. 2016). However, the same does not apply to the majority of monkey species. Unlike great apes, for which population density can be estimated making use of their habit to build sleeping platforms called “nests” (Tutin and Fernandez 1984), other primate species do not leave obvious signs of their presence and must be monitored by direct observation or by their vocalizations (Plumptre et al. 2013). Nevertheless, many of these species are also decreasing as a result of habitat destruction (Cavada et al. 2019; Remis and Jost Robinson 2012) and over-hunting (Kümpel et al. 2008; Linder and Oates 2011; Peres 1999; Rosenbaum et al. 1998). In Central Africa, primate habitat loss is accelerating due to (1) small-scale slash-and-burn agriculture of increasing rural population (Tyukavina et al. 2018), as well as (2) widespread extraction of natural resources like minerals and timber driven by increasing global demand (Abernethy et al., 2016). The problem is exacerbated by hunting pressure. In fact, primates are among the most affected species, being targeted by both commercial (Bachand et al., 2015; van Vliet et al. 2012) and subsistence hunters (Fa et al. 2016).

The debate on best practices for the assessment of arboreal primate population status is still ongoing. Primates inhabit highly diverse habitats, spanning open savannahs, rainforest, and mountain ranges, and differ significantly in terms of ecology and behavior. As a result, the survey methods suitable for a species in a given habitat, may not provide accurate or precise density estimates in other contexts. To overcome the complexity of surveying arboreal primates in the wild, depending on the habitat and species of interest, recent methods suggested the application of acoustic playback (Gestich et al. 2017), passive acoustic monitoring (Kalan et al. 2015), camera traps (Bessone et al. 2020; Moore et al. 2020), and drones (Semel et al. 2020; Spaan et al. 2019). However, these novel techniques are still under development and cannot be applied to all species (e.g., acoustic methods can only be used for highly vocal species), leaving line transect distance sampling (LTDS) as the method of choice for surveying primates (Buckland et al. 2001; Plumptre et al. 2013). LTDS consists of linear sample units, called transects, paths walked by trained observers to count primates in vision. Being based on direct observations, LTDS has the advantage of being applicable to any species. As each observed primate is recorded along with its perpendicular distance from the transect, LTDS allows estimating density by modeling the probability of observing a monkey as a function of distance. In short, the further a monkey is from the transect, the lower the probability of spotting it. By modeling detection probability, the application of distance sampling provides unbiased estimates of the true population density in the study area, conditional on sufficient survey effort and the deployment of an adequate number of transects (*n* > 20, i.e., replication) placed randomly throughout the study area (i.e., randomization), as well as the fulfilment of certain assumptions (Buckland et al. 2001).

In the past, however, the application of LTDS to primates raised concerns (Chapman et al. 2010; Hassel-Finnegan et al. 2008; Marshall et al. 2008). Some studies reported large overestimates of the true primate density (Chapman et al. 2010; Defler and Pintor 1985; Hassel-Finnegan et al. 2008), while others showed the opposite (Cavada et al. 2017a, b; Skorupa 1987). Mostly, the reported biases were based on poor survey designs, with studies lacking adequate effort, replication, and randomization (Buckland et al. 2010a). However, even when surveys are carefully designed, surveying primates remains challenging. In order to obtain reliable density estimates, three main issues, along with their related assumptions, must be thoroughly considered during data collection and analysis.

*1) Group size*. As social mammals, most arboreal primates range and feed in groups up to several dozens of individuals. Because measuring distances to each individual within a group is, albeit preferable, rarely feasible, LTDS usually considers observation of groups rather than individual monkeys (Marshall et al. 2008; Plumptre and Cox 2006). Abundance in the area is then estimated as a function of group size, which must be accurately recorded (assumption 1). In the field, however, primate group size is difficult to assess, particularly in tropical forest where group members are scattered and hidden in the canopy (Araldi et al. 2014; Ferrari et al. 2010). While inaccurate estimation of group size far from the transect line is not expected to cause bias, it is crucial that at least the size of groups close to the line is accurately estimated (Buckland et al. 2010a).

*2) Group spread*. To correctly estimate the detection probability, LTDS requires the measurement of the perpendicular distance of each observed group to the transect line. Perpendicular distances need to be measured to the group center, and it is assumed that groups with their center approximately on the transect line, are detected with certainty (assumption 2). Furthermore, LTDS assumes that perpendicular distances are measured accurately (assumption 3). Therefore, to locate the group center, observers must first define the spread of the group. As this is not a trivial task (Buckland et al. 2010a, b; Chapman et al. 2010; Marshall et al. 2008), methods improving standard applications have been recently proposed (Cavada et al. 2017a, b).

3) *Reactivity to the observer.* Finally, LTDS requires that groups must be detected before any response to the observer, i.e., before they flee (assumption 4). Violation of assumption 4 would bias the estimated density by prohibiting observers from correctly detecting groups, eventually constraining the accuracy of the detection function. In tropical forests, the issue is exacerbated by the thick understorey causing disturbance when walking, but more importantly requiring observers to cut their line transects. In addition, primates may react by avoiding areas previously visited by the researchers, e.g., where transects have been cut recently. There are different practical suggestions to minimize disturbance such as (a) reducing the noise of cutting by using secateurs rather than machetes (Buckland et al. 2010a); (b) cutting the transect a few days before the actual survey (Plumptre et al. 2013), e.g., 7 days (Araldi et al. 2014; Hofner et al. 2020). However, to our knowledge, studies investigating the effect of disturbance on counts, including recommendations as of the time-lag needed between transect cutting and actual count, are absent.

In this study, we investigated the effect of primate reactivity to the observer. We tested the effect of the time between the disturbance “transect cutting” using machetes, and the actual count on (a) encounter rates (ER), i.e., number of groups per km; (b) observed group size (GS), i.e., number of individual primates within a group; (c) estimated densities (d), i.e., number of individuals per km^2^; and (e) species-specific differences in (a), (b), and (c).

To do so, we applied LTDS in Salonga National Park (SNP), Democratic Republic of the Congo (DRC), the largest protected forest area of the African continent, to five primate species: Tshuapa red colobus (*Piliocolobus tholloni*), Angolan colobus (*Colobus angolensis*), Black mangabey (*Lophocebus aterrimus*), red-tailed monkey (*Cercopithecus ascanius whitesidei*), and Wolf’s monkey (*Cercopithecus wolfi*). The population of these species are considered decreasing in the wild, with four of them being classified as vulnerable or endangered: *P. tholloni* (VU), *C. angolensis* (VU), *L. aterrimus* (VU), *C. crysogaster* (EN), *C. wolfi* (NT) (IUCN 2020).

## Methods

### Study area

Salonga National Park (36,000 km^2^) is an UNESCO World Heritage Site situated in DRC. It is formed by two blocks, North and South, separated by an inhabited corridor (9000 km^2^). We investigated the block South (17,127 km^2^), composed 99% of primary lowland mixed forest, 1% of savannahs, regenerating forest, cultivation, marshes and water bodies (Bessone et al. 2020). Nine diurnal primate species are known to be present in the park. In addition to those mentioned above, SNP harbors the endangered Golden bellied mangabey (*Cercocebus crysogaster*), Allen’s Swamp monkey (*Allenopithecus nigoviridis*), De Brazza’s monkey (*Cercopithecus neglectus*), and a great ape, the bonobo (*Pan paniscus*).

### Data collection

#### General design

**Fig. 1:**
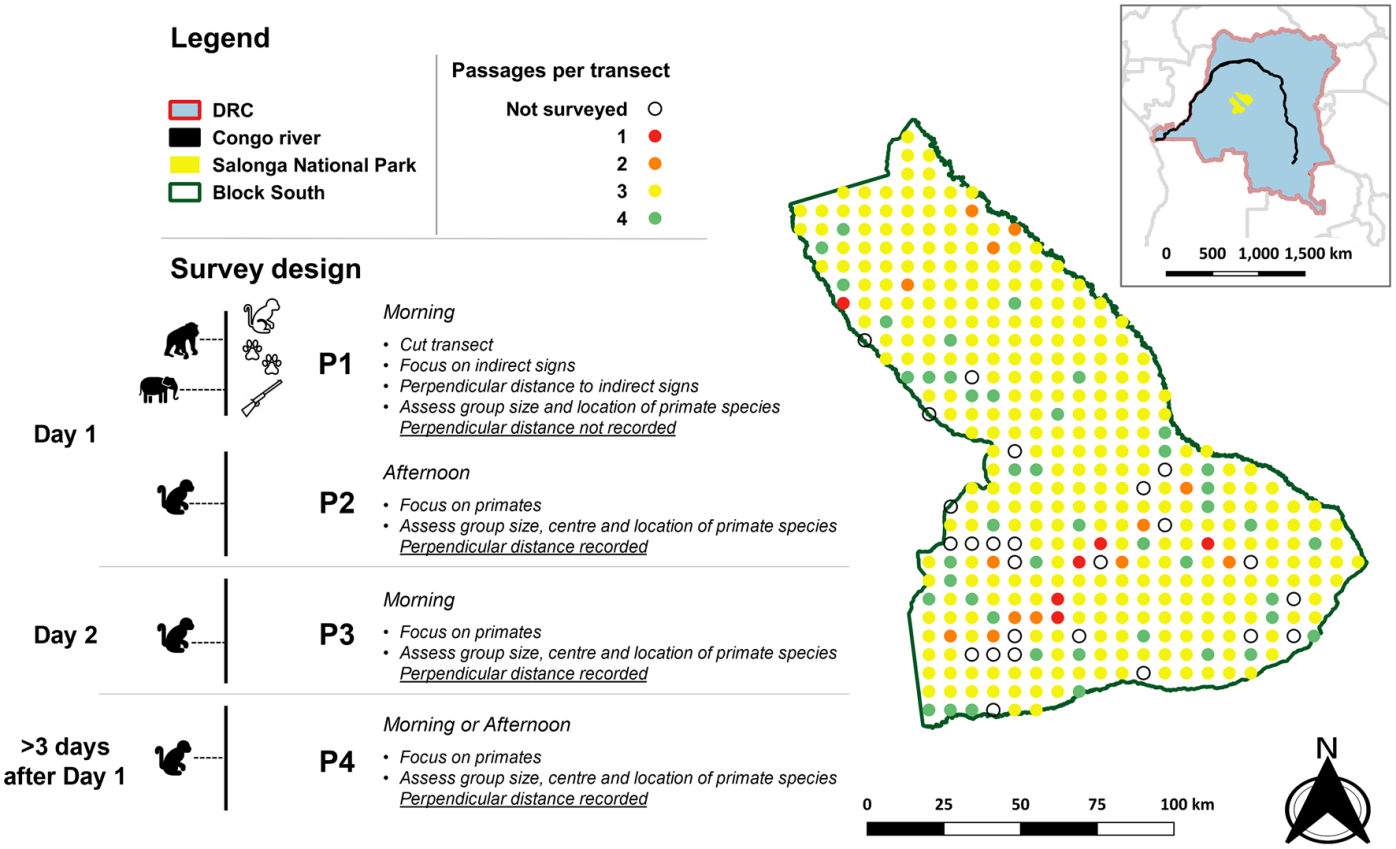
Study area and survey design

LTDS data were collected between September 2016 and May 2018 as part of a comprehensive biodiversity inventory (PNS-Survey©). The survey consisted of 405 transects and was designed in Distance 6.0. (Thomas et al., 2010), with transects starting from a random origin and then placed systematically in the study area. Each transect was 1 km long, spaced from other transects by 6 km in both the east–west and north–south directions (Fig. 1). This design allowed us to (1) obtain a uniform coverage of the study area (Thomas et al., 2010) and (2) survey one transect and reach the next one in a single day. As in our study, five monitoring teams surveyed 6–8 transects per month (i.e., one survey block per month) independently in different areas, the latter was logistically important, as it allowed us to complete a survey block within the planned timeframe.

Each transect was walked 3–4 times. The number of passages (i.e., repeated transect walks), was a trade-off between (1) the need to investigate how survey-inherent disturbance affected primate counts at different times after the first disturbance event, and (2) the feasibility of the study in terms of logistics and time.

To minimize disturbance, we walked in teams of four members only, without carrying loads except for the equipment needed for observing wildlife, measuring perpendicular distances, and recording data. To increase the probability of spotting all primate groups present on the transect, we walked the transects silently and no faster than 0.6 km per hour, minimizing the chances of startling the groups. We also did not walk the transects in the rain, when primates are less active, and did not mark the starting and ending point of the transect in any way visible to the primates. In subsequent passages, we recognized the transect starting point by using its GPS location (marked during the first passage), while the transect line was identified by the presence of (1) a Topofil® thread and (2) vegetation cuts left during the previous passage.

During passage 1 (P1), each transect was opened. To minimize disturbance, we used secateurs whenever possible, although machetes were allowed to be used when required by the thickness of the understory. During P1, one observer was tasked with cutting the transect, while the other three were only required to observe wildlife and wildlife’ signs. We assumed P1 to cause the highest disturbance level and focused on counting indirect signs of sympatric wildlife such as droppings, nests, tracks, and human activity signs rather than on directly observing primates. If a monkey group was observed during P1, species and group size were recorded, but not its distance to the transect. Thus, P1 was not used for the estimation of primate density. P1 was performed in the morning, not before 7:00 am.

Passage 2 (P2) was walked on the same day of P1, and it was the first passage where we recorded perpendicular distance to observed primate groups. It was performed in the afternoon, not before 3:00 pm, as primates are mostly active in the morning and in the late afternoon. In addition, to allow a few hours for the monkeys to recover from potential disturbance, minimum time-lag to completion of P1 was 3 h (N’Goran et al., 2016. By that, we assumed P2 to have the second-highest level of disturbance.

Passage 3 (P3) was performed the day after P1 and P2 and also focused on primate counts. It had to occur in the morning (from 7:00 am) but was postponed to the afternoon (after 3:00 pm) in case of morning rain. We considered P3 to allow even more time for the monkeys to readjust, and thus to have the third-highest level of disturbance. After P3 was completed, the monitoring teams immediately moved to the next transect, in order to reach it before dusk.

Passage 4 (P4) also focused on primate counts but was performed a minimum of 3 days after P1 was completed, either at 7:00 am or after 3:00 pm. P4 had the purpose to assess the influence of disturbance on the detection of monkey groups. For logistical reasons, this passage was not aimed to be conducted on all (*n* = 405) transects but on a selection of at least 10% (*n* = 41). To achieve this percentage, we selected one transect per survey block consisting of 6–8 transects each. We assumed P4 to have the lowest level of disturbance.

When we heard a primate group vocalizing, we recorded the point on the transect from where we first heard the group and estimated its distance to the transect and identified the species from its vocalization. Here, as we could not directly observe them, we were unable to identify poly-specific groups with certainty and thus considered each species as mono-specific group. The estimated distances to the transect were then recorded as (a) “close”, if assumed to be closer that 100 m; (b) “far” if estimated being between 100 and 500 m; and (c) “very far” if further than 500 m.

When we spotted a primate group, all observers left the transect line to determine group (a) size, (b) spread/center, and (c) perpendicular distance to the transect line.

#### Determining group size

We recorded two different values of group size: (1) observed, i.e., how many individual primates were directly spotted; (2) estimated, i.e., how many individual primates were inferred to be present by adding individuals heard but not seen to those observed. Each team member recorded observed group size (1) resulting in four independent counts. In case of poly-specific groups, we counted the number of individuals of each species. For each species, we retained the highest value observed. The same was done for the estimated group size (2). Here, to control for extreme estimated values and/or the risk of consistent upward bias due to the involvement of different observers, we retained the average of the estimated group sizes (*n* = 4) rather than the highest value.

#### Estimating group spread

When determining group spread, we focused on observed individuals only (N’Goran et al., 2016). If the group was at one side of the transect only (1), we estimated group spread by determining the position of the closest and furthest individuals observed. However, if the group was at both sides of the transect (2), we estimated group spread by detecting the position of the furthest individuals observed at each side. In both cases, we defined the group center as the midpoint between the two individuals. In case of poly-specific groups, we used the same point for each species.

#### Measuring perpendicular distances

When we defined the location of the group center, we measured (measuring tape) the perpendicular distance from the group center (in centimeters) to the transect line (marked by a Topofil® thread) following the principles of distance sampling (Buckland et al., 2010a). Finally, we marked the point on the transect perpendicular to a group’s center using a handheld GPS (Garmin 64S) and recorded all additional information on a smartphone (Samsung Galaxy XCover 3) using the Cybertracker software (ver. 3.435).

All team members were trained in LTDS techniques, including (1) compass/GPS navigation; (2) transect cutting and walking, (3) primate group size, spread and perpendicular distance estimation, (4) data recording using the Cybertracker software (N’Goran et al. 2016), during theoretical and practical workshops conducted (1) August 2016 (Monkoto, Tshuapa, DRC) and (2) September 2017 (Mundja, Mai Ndombe and Anga, Kasai, DRC) (Bessone et al. 2019).

### Data analysis

We tested for specific differences in encounter rates, i.e., group observed or heard per kilometer, between passages by comparing the average encounter rate (ER) of (a) all species taken together and (b) each primate species. To do so, we modeled ER in each transect *i*

$$ER_{i} \sim {\text{~Negative~Binomial}}\left( {\eta _{r} ,~\varphi _{r} } \right)$$

where η_*r*_ is the mean encounter rate in passage *r,* and φ is the overdispersion parameter for passage *r*. The negative binomial distribution fitted our dataset, as 46% of transects had no observations (i.e., ER = 0) and a few transects had many observed groups.

Similarly, we checked for differences in species-specific observed group size, i.e., number of individuals per group, between passages by comparing the mean observed group size (GS) in each transect *i*

$$GS_{i} \sim {\text{~Lognormal}}\left( {\mu _{r} ,\sigma _{r} } \right)$$

where μ_*r*_is the mean group size and σ_*r*_ is the standard deviation in passage *r.* Here, the lognormal distribution was used to fit a dataset of positive values only. In this analysis, we only considered transects where we had at least one observed group (i.e., GS > 0) and excluded observations made in P1. By that, we avoided bias due to a possible lack of attention in assessing GS in P1, where monkey counts were not the focus.

We coded our models using the package RStan ver. 2.26.11 (Stan Development Team, 2020) in R 4.1.1 (R Core Team, 2021). For each model, we ran four chains of 2000 iterations (1000 warm-up).

We estimated primate density using Distance 7.3 for Windows (Thomas et al., 2010). We first fitted a single detection function for the aggregated analysis of all species together, and then species-specific detection functions to estimate specific densities per passage. For each analysis, we first right-truncated the data following the visual inspection of the histogram of observed distances. Then, we compared models derived from all possible combinations of key function (i.e., half normal; hazard rate; uniform), series expansion (i.e., cosine; simple polynomial) and adjustment terms (i.e., from 0 to 3), selecting the best-fitting model according to lowest Akaike information criterion (AIC). We considered group size in our analyses by estimating an average group size for each species. To do so, we regressed the natural logarithm of group size against distance if the significance of the regression was significant at an alpha level of 0.1. By that, we aimed to reduce the bias induced by smaller groups being missed at larger distances more often than large groups. However, if the regression was not significant, the average group size was equated to the observed average size, i.e., no regression was used (Thomas et al., 2010). As we wanted to highlight differences in estimated density between passages for different species, we performed density analyses for all datasets for which it was possible to obtain a good fitting of the detection function.

However, in order to provide the most reliable density estimates, we calculated a “corrected density” by (1) only considering the passages providing the highest encounter rates; (2) correcting the resulting estimates of group density by the highest average estimated group size obtained within passages. In practice, we did not consider passages providing significantly lower encounter rates when calculating the corrected density (Table 1). Similarly, as we assumed that the highest estimated group size between passages was closer to the real group size, we multiplied the estimated number of primate groups (for each species) obtained by the largest estimated group size returned from passage specific Distance analyses (Table 2).

**Table 1:** Descriptive statistics by passage

Passage	Transects (*n*)	Morning transects (%)	Effort (km)	Encounter rate (groups/km)	Encounter rate of poly-specific groups (*n*/km)	Perpendicular distance (cm)	Estimated distance of heard groups (*n*)		
							< 100 m	100–500 m	> 500 m
P1	378	0.963	374.17	0.935	0.037	*NA*	122	102	5
P2	358	0.000	354.63	0.936	0.054	1684	46	57	3
P3	371	0.992	367.46	1.230	0.086	1660	100	68	5
P4	51	0.569	51.02	1.196	0.118	1751	13	4	0
Total/*Average*	**1158**	*0.631*	**1147.28**	*1.004*	*0.074*	*1698*	**281**	**231**	**13**

Transects (*n*): number of transects surveyed; Morning transects (%): proportion of transects surveyed in the morning; Effort (km): total length of walked transects; Encounter rate (groups/km): number of primate groups encountered (observed and heard) per km; Perpendicular distance (m): average perpendicular distance in meters to center of observed groups; Estimated distance of heard groups (*n*): number of primate groups heard vocalizing (1) within 100 m, (2) between 100 and 500 m, (3) beyond 500 m. The bottom line provides totals (*bold*) or averages (*italics*)

**Table 2:** Estimated primate density (individuals/km^2^) by passage and species using (1) observed and (2) estimated group size

Species	Density (ind/km^2^) [95% CI] Group size [SD, *n*]					
	P2		P3		P4	
	Observed	Estimated	Observed	Estimated	Observed	Estimated
*L. aterrimus*	**52.23 [33.10–82.41]**	**68.34 [43.65–107.01]**	**61.32 [38.72–97.12]**	**79.36 [48.45–129.98]**	**52.13 [27.88–97.49]**	**75.15 [28.38–198.97]**
	8.58 [8.89, *76*]	11.24 [10.83, *76*]	9.4 [8.19, *90*]	12.29 [9.81, *90*]	12.59 [7.62, *17*]	16.53 [10.03, *17*]
*P. tholloni*	**25.27 [12.35–51.73]**	**39.94 [19.45–82.05]**	**69.31 [43.07–111.54]**	**90.46 [55.81–129.98]**	**/**	**/**
	18.65 [13.43, *26*]	28.65 [21.54, *26*]	22.27 [12.62, *40*]	30.88 [17.88, *40*]	16.67 [10.98, *6*]	22.17 [12.92, 6]
*C. angolensis*	**2.97 [1.51–5.86]**	**4.12 [2.01–8.49]**	**4.5 [2.43–8.34]**	**5.4 [2.76–10.55]**	**/**	**/**
	2.40 [1.18, *15*]	3.4 [2.23, *15*]	3.00 [1.55, *25*]	5.08 [3.44, *25*]	1.5 [0.71, *2*]	1.5 [0.71, *2*]
*C. ascanius*	**13.73 [9.14–20.62]**	**20.19 [13.31–30.62]**	**36.5 [27.84–47.84]**	**50.23 [38.47–65.60]**	**56.92 [25.49–127.11]**	**68.31 [30.78–151.60]**
	5.38 [4.70, *69*]	7.45 [5.97, *69*]	6.67 [5.14, *110*]	9.30 [6.68, *110*]	8.22 [4.73, *18*]	10.83 [4.59, *18*]
*C. wolfi*	**8.32 [4.53–15.29]**	**10.84 [5.91–19.82]**	**19.52 [12.07–31.58]**	**30.39 [18.94–48.76]**	**/**	**/**
	6.79 [5.12, *19*]	9.32 [6.49, *19*]	6.43 [5.59, *49*]	9.88 [8.11, *49*]	8.29 [3.45, *7*]	13.14 [8.28, *7*]
*All species*	**112.45 [81.84–154.52]**	**147.57 [105.69–206.40]**	**196.87 [155.87–248.67]**	**277.71 [220.00–350.55]**	**230.1 [147.10–359.93]**	**311.55 [181.63–534.41]**
	8.16 [8.96, *205*]	11.42[12.82, *205*]	9.11 [9.03, *314*]	12.66 [12.08, *314*]	10.46 [7.25, *50*]	14.08 [9.28, *50*]

For each passage and species, the mean estimated density (*bold*) with confidence interval (*in squared brackets*) by group size method. Observed: only individuals seen are considered; Estimated: individuals localized acoustically are included

## Results

Due to logistical constraints (i.e., accessibility, safety), we conducted primate counts on 378 transects (out of 405 planned), including repeated passages on the same transects (*n* = 1158). Due to rain in the afternoon, P2 was conducted on 358 transect only, while the presence of armed poachers (*n* = 1) and the flooding of the transect area after heavy rain (*n* = 6) prevented conducting P3 on seven transects, which was thus only conducted on 371 transects. The resulting total effort was 1147.28 km of transects. Of these, 66% (*n* = 761) were walked in the morning, while the remaining 34% (*n* = 397) were walked in the afternoon (Table 1). P4 was conducted on 13% of surveyed transects (*n* = 51), 10.33 days on average (SD = 5.88) after the transect cutting (P1). Here, 53% (*n* = 29) of transects were walked in the morning and 47% (*n* = 22) in the afternoon.

### Encounter rates (ER)

We encountered a total of 1153 primate groups resulting in an average encounter rate of one group per kilometer (Table 2), including re-sightings of the same group (as same transects were surveyed multiple times). Of these, 1085 groups were mono-specific, and 68 were poly-specific, including two (*n* = 57) or three (*n* = 11) different species. We observed fewer poly-specific groups per kilometer in P1. Encounter rates increased with reduced disturbance, with P4 showing three times higher encounter rates of mixed groups than P1 (Table 1). The average observed group size consisted of 8.89 (SD = 8.87) individuals, the average estimated group size of 12.34 individuals (SD = 12.14). Estimated groups sizes were 39% larger than observed ones.

In addition, the total number of encounters included 525 groups only heard vocalizing (i.e., not observed), 97% of which were estimated being within 500 m from the transect line. Here, P2 showed a larger proportion of “far” vocalizations (54%) than other passages (P1 = 44%; P3 = 39%; P4 = 24%) (Table 1). There was a trend to differences being significant between P2 and P3 (*X*^2^ = 5.678,* p* = 0.055) as well as P2 and P4 (*X*^2^ = 6.524,* p* = 0.064) when performing pairwise *X*^2^ tests between passages. All other comparisons were not significant (*p* > 0.1).

**Fig. 2:**
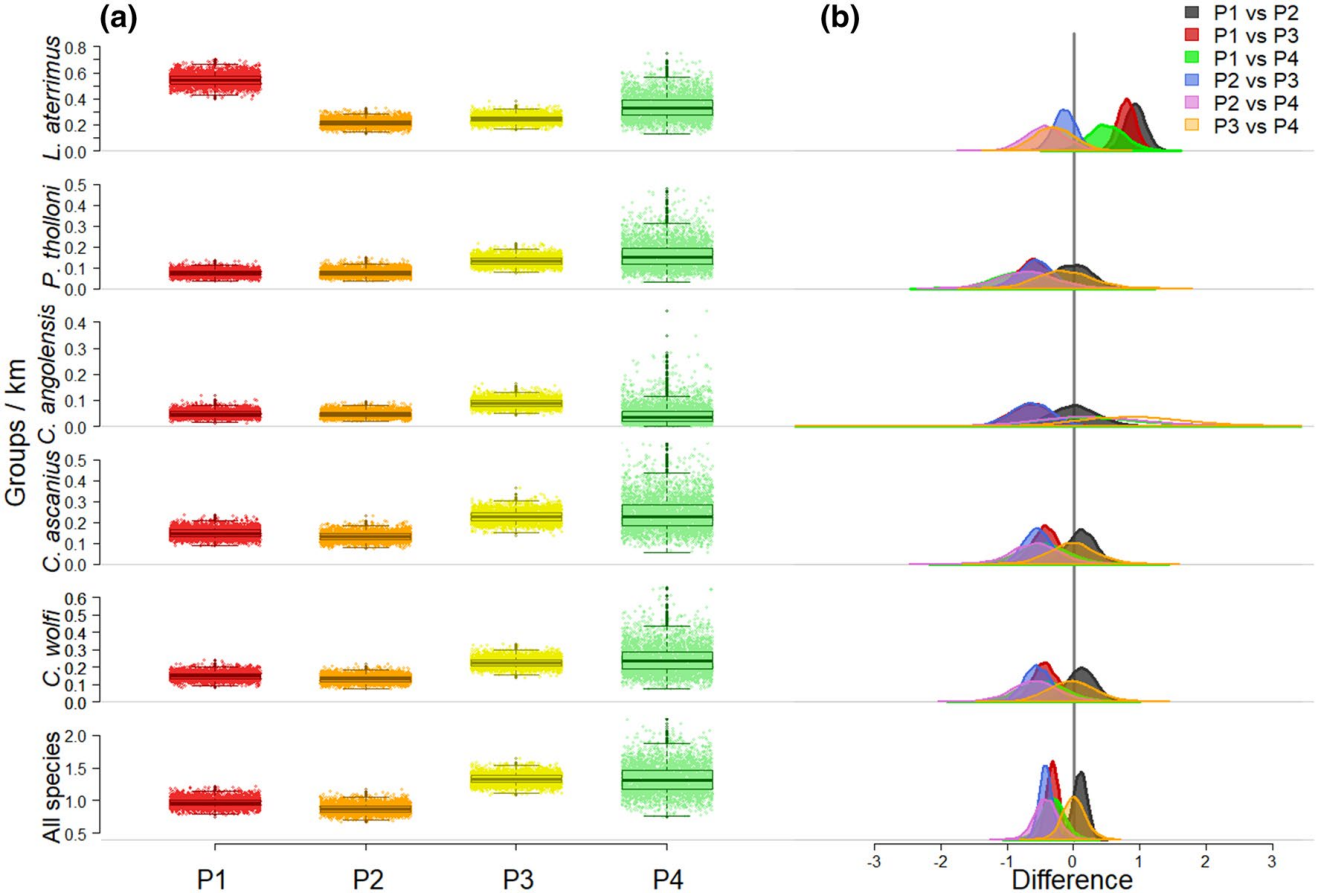
Differences in encounter rates between passages and species.** a** Average encounter rate for each passage (P1 = *red*; P2 = *orange*; P3 = *yellow*; P4 = *green*) by species separated (rows 1 to 5) and together (*bottom row*);** b** posterior distribution of pairwise contrasts of average encounter rates between passages for each species

When testing for differences in encounter rates (ER) between passages, for most species rates consistently increased with reduction of disturbance, with the highest ER found in P3 and P4 (Fig. 2). This did not apply to the black mangabey *L. aterrimus,* for which we observed the highest ER in P1 (highest disturbance), followed by P4 (lowest disturbance). The black mangabey was also the species with the highest proportion of heard groups during P1, with 76% of encounters being vocalizations rather than direct observations. This percentage decreased to 32% in P4 (Supporting Table 1).

### Group size (GS)

**Fig. 3:**
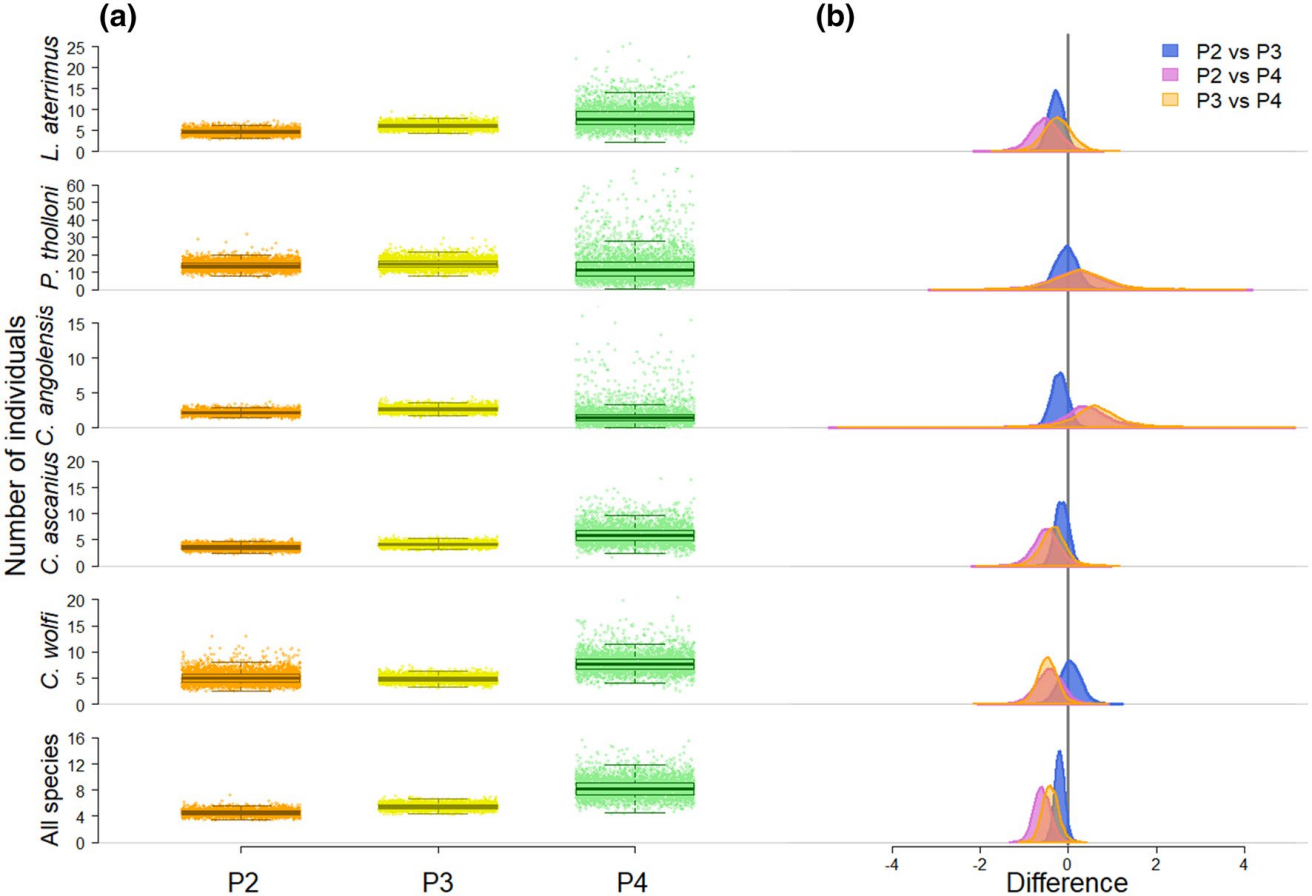
Differences in observed group size between passages and species.** a** Average group size observed in each passage (P2 = *orange*; P3 = *yellow*; P4 = *green*) by species (rows 2 to 6) and all species together (*top row*);** b** Posterior distribution of pairwise contrasts of average observed group size between passages for each species

The analysis of the differences in group size between passages revealed a similar pattern, with observed group size being higher in P4 with the lowest disturbance. Here, the main exception was represented by the red colobus *P. tholloni*, showing group size consistent between passages (Fig. 3). Similar results were obtained with estimated group size (Supporting Fig. 1), with the two measures being highly correlated (Spearman rank correlation test: rho = 0.94,* p* < 0.001).

### Density estimates (D)

Calculated densities increased considerably with a reduction in disturbance (Table 2), with P2 returning the lowest estimates for all species. The limited number of observed groups in P4 did not allow to fit a detection function and calculate a density value for three species: *C. tholloni* (*n* = 6), *C. angolensis* (*n* = 2) and *C. wolfi* (*n* = 7). In addition, due to the small number of transects and observed groups in P4 (Supporting Table 1), confidence intervals (CI) associated with densities the two species with sufficient observations, *L. aterrimus* and *C. ascanius*, were very large. Here, the 95% CI obtained in P4 completely overlapped the one obtained in P3. However, the black mangabey was again an exception, showing estimated densities consistent between passages (Table 2). The use of estimated group size consistently increased estimated densities for all species and across all passages by 36% on average (min = 20%, max = 58%) (Table 2).

Following the results above, we also calculated the corrected estimate for each species. Here, we included all observations from P3 and P4, showing consistent ER, discarding those obtained in P2. Then we corrected for group size by using the largest average estimated size obtained. Consequently, we used estimated size observed in P3 for *P. tholloni* and *C. angolensis*, and corrected by estimated size obtained in P4 for *L. aterrimus*, *C. ascanius* and *C. wolfi* (Table 2).

By that, we obtained densities of (i) 90 ind/km^2^ (95% CI = 61–134) for *L. aterrimus*; (ii) 99 ind/km^2^ (61–161) for *P. tholloni*; (iii) 7 ind/km^2^ (4–12) for *C. angolensis*; (iv) 62 ind/km^2^ (45–85) for *C. ascanius*; and (v) 43 ind/km^2^ (26–70) for *C. wolfi*, similar to other sites (Table 3). We provide a detailed description of these analyses, including sample size, selected models, and plots of fitted detection functions of the corrected analyses, in Supporting Table 2.

**Table 3:** Comparison of published densities and group size to corrected estimates (this study)

Species	Group size mean (range)		Density mean (95% CI)		Method group size-density		Reference	Site
*L. aterrimus*	16.5 (1–60)		90.1 (60.7–133.7)		ES	DS	*1*	Salonga NP (DRC)
	/ (10–11)		73.1 /		ES	AO	*2*	Lomako (DRC)
	/ (14–19)		69.0 /		FG	HR	*3*	Lake Tumba (DRC)
	/ (10–11)		/ /		FG	/	*4*	Salonga NP
*P. tholloni*								
	30.9 (1–100)		99.2 (61.0–161.2)		ES	DS	*1*	Salonga NP (DRC)
	60.0		24.0 /		FG	HR	*5*	
	13.2 (/–45)		/ /		FG	/	*6*	
	29.9 (5–63)		/ /		FG	/	*6*	
*C. angolensis*	4.0 (1–16)		6.7 (3.6–12.4)		ES	DS	*1*	Salonga NP (DRC)
	5.0 /		5.8 /		FG	ST	*2*	Lomako (DRC)
	/ (6–20)		16.7 (5.3^§^)		FG	SW	*7*	Ituri (DRC)
	/ (6–16)		7.7 (4.0^§^)		ES	DS	*8*	
	/ (3–7)		/ /		FG	/	*5*	Salonga NP (DRC)
	/	/	/	(2.9–12.0)	ES	DS	*9*	Udzungwa (DRC)
(Tanzania)								
	/	(2–14)	/	/	FG	/	*10*	
*C. ascanius*	10.8	(1–36)	62.2	(45.2–85.8)	ES	DS	*1*	Salonga NP*(DRC)
	14.7	/	42.8	/	ES	ST	*2*	Lomako*(DRC)
	/	(3–11)	18.9	(4.4^§^)	ES	DS	*8*	Ituri^**^(DRC)
	/	(17–23)	117.0	/	FG	HR	*11*	Bangui^**^(CAR)
		(13–18)	46.4	/	FG	DS	*12*	Budongo^**^(Uganda)
	13.8	(12–18)	8.3	/	FG	DS	*12*	
	15.6	(13–18)	60.0	/	FG	PD	*13*	
	13.8		19.2	/	FG	PD	*13*	
	/	(14–16)	38.1	/	FG	AO	*14*	Kibale^**^(Uganda)
	/	(27– 29)	135.1	/	FG	AO	*14*	
	/	(30–35)	/	/	FG	/	*15*	
	/	(28–35)	/	(140.0–175.0)	FG	AO	*16*	
	/	(14–35)	131.5		FG	AO	*17*	
	/	(19–29)	/	(70.0–158.0)	FG	HR	*18 asis>*	
	32.0		162.0	/	ES	AO	*19*	
	/	(25–50)	/	/	FG	/	*20*	
	/	(10–35)	/	/	FG	/	*21*	
*C. wolfi*	13.1	(1–50)	43.1	(26.5–70.0)	ES	DS	*1*	Salonga NP (DRC)
	10.1		44.0	/	ES	AO	*2*	Lomako (DRC)
	16.0	(13–25)	30.0	/	FG	HR	*4*	Salonga NP (DRC)

“Species”: species of interest, populations from forest habitats only (e.g., woodland-savannah sites are omitted); “Groups size”: mean and minimum-maximum (in brackets) reported group size; “Density”: mean and 95% confidence intervals (unless differently specified, in brackets) reported population density; “Method”: method used for 1) reported group size; i.e. “ES” = estimated group size from line transect observations; “FG” = direct observation of focal groups and 2) reported population density i.e. “AO” = strip transect, with strip width defined using the maximum reliable sighting distance measured as the distance from where a primate group was first sighted to the group itself, i.e. the “animal-observer distance; “DS” = line transect distance sampling; “HR” = number observed individuals / observed home range size; “PD” = strip transect, with strip width defined as the maximum reliable sighting distance measured using perpendicular distances to the primate group centre; “SW” = sweep counts); References: [1] This study, [2] McGraw 1994, [3] Horn 1987, [4] Gauthier-Hion 2013, [5] Maisels et al. 1994, [6] IUCN 2021, [7] Bocian 1997, [8] Thomas 1991, [9] Araldi et al. 2014, [10] Rovero et al. 2006, [11] Galat-Luong 1973, [12] Plumptre and Reynolds 1994, [13] Sheppard 2000, [14] Chapman and Lambert 2000, [15] Struhsaker 1975, [16] Struhsaker 1980, [17] Struhsaker 1978, [18] Struhsaker 1997, [19] Mitani et al. 2001, [20] Windfelder and Lwanga 2004, [21] McLester et al. 2019; “Site”: area and country where the study took place. *Cercopithecus ascanius whitesidei; ** Cercopithecus ascanius schmidti; § standard error

## Discussion

Our study used 1147.28 km of 1-km line transects evenly spread over 17,127 km^2^ of pristine lowland rainforest, to investigate how survey-inherent disturbance affected primate detectability, hence the accuracy of resulting estimates of density and abundance. We found that survey-inherent disturbance had a negative effect on both encounter rate (ER) and observed group size (GS), leading to underestimated densities when applying LTDS. This is an important finding with far-reaching consequences, which, to our knowledge, has received only little attention by both practitioners and researchers.

### Encounter rates (ER)

The number of encountered groups per km of transect (ER) was lowest the day the transect was opened (P1), remaining consistent for a few hours (P2). The ER increased substantially in P3, only 24 h after the highest disturbance in P1, and remained stable in P4, occurring a minimum of 72 h after P1 (Fig. 2). By that, our results suggest that 24 h were sufficient for the primate groups to regain their usual routine and area of activity after disturbance. However, the black mangabey *L. aterrimus* was an exception, as we detected the highest ER in P1, concomitant with the transect opening. The slight increase in ER observed with decreasing disturbance (from P2 to P4, Fig. 2), never approached the rates observed in P1, the passage with the highest disturbance. While our results seem to suggest that *L. aterrimus* required longer than any other species to re-occupy the areas where they had been disturbed, another more obvious explanation for this unexpected result may be the mangabey’s vocal behavior. *L. aterrimus* produces long-distance calls (up to 1 km in the allopatric grey-cheeked mangabey (*Lophocebus albigena)* (Brown 2020) used to signal (a) inter-group spacing and intra-group rallying, i.e., “whoop-gobble” and (b) alarm, i.e., *karaou* (Kingdon et al. 2013). As confirmed by the high proportion of heard vs. observed groups in P1 (76%), black mangabeys were more frequently heard calling during transect cutting (Supporting Table 1). Although the frequency of whoop-gobbles should not have been affected, we expect mangabeys to emit fewer alarm calls in passages with reduced disturbance (Campos and Fedigan 2014). This hypothesis could have been tested by investigating between-passages differences in call type frequency, which was not recorded in our study. Conversely, red colobuses and red-tailed monkeys were the least vocal (Supporting Table 1). Like in our study, Western red colobuses (*Piliocolobus badius*) in Taï National Park, Cote d’Ivoire, were found being relatively silent when spotting a potential predator (i.e., chimpanzee), taking advantage of Diana’ monkeys (*Cercopithecus diana*) as sentinels (Noë and Bshary, 1997). Similarly, in Taï, five primate species, including Western red colobuses *(P. badius)*, Western black and white colobus (*Colobus polykomos*), Diana’s monkey (*C. diana*), the lesser white nosed monkey (*Cercopithecus petaurista*), the Campbell’s guenon (*C. campbelli*) and the sooty mangabey (*Cercocebus atys*), were found to emit fewer alarm calls when spotting a chimpanzee (thus avoiding signaling their presence), than when spotting a leopard (Zuberbühler et al. 1999). However, the opposite was observed in Kibale National Park, Uganda, where Ashy red colobuses (*Piliocolobus tephrosceles*) were extremely vocal when spotting a chimpanzee (Stanford, 1995). In SNP, *P. tholloni* were the most targeted species by poachers in the past (Thompson 2000). As a result, they might have responded to high hunting pressure from humans by reducing their alarm call rate, avoiding signaling their presence, like species such as the golden-bellied capuchin (*Sapajus xanthosternos*, Suscke et al. 2021) and the woolly monkey (*Lagothrix poeppigii*, Papworth et al. 2013).

Finally, we also observed a higher number of poly-specific groups with reduced disturbance (Table 1), consistent with other observational studies, where species associated to increase foraging and anti-predatory efficacy (Teelen, 2007), but split immediately when threatened (Stanford, 2002). In sum, the response to disturbance was species-specific, highlighting the need to consider site-specific behavioral and ecological features.

### Group size (GS)

Our results showed that disturbance affected observed species’ group size (GS). As with the encounter rate, GS increased with time to transect cutting, with large group sizes being recorded in P4. However, a difference was still noticeable between P3 and P4, suggesting that a pause of 24 h to the initial cut were not sufficient to observe all individuals (Fig. 3). At the species level, this pattern was consistent in* L. aterrimus*,* C. ascanius*, and* C. wolfi*, all belonging to the sub-family Cercopithecinae. In contrast, the two Colobinae, * P. tholloni* and *C. angolensis*, showed no noticeable difference between passages, although the low number of *C. angolensis* observations in P4 (*n* = 2) did not allow any meaningful comparison.

These results can be explained by the ecological and behavioral differences between the Cercopithecinae and Colobinae sub-families. For example, red colobus monkeys are a highly social species, living in groups of up to 60 individuals (Maisels, et al., 1994), taking advantage of group cohesiveness as an antipredator strategy. In Kibale, Uganda, red colobus (*P. tephrosceles*) groups did not flee when noticing a predator, i.e., chimpanzees (Stanford 1995). Instead, females grabbed their juveniles and approached the adult males, standing their ground to the predator (Stanford 1995). As such, their naivety often reported when encountering humans (e.g., Nowak et al. 2016) could be the manifestation of this very anti-predatory behavior. Alternatively, the difference observed between sub-families could be triggered by anatomy: cheek pouches specific to Cercopithecinae are lacking in Colobinae. Cheek pouches allow to store and transport food for later ingestion somewhere safe in the canopy (Lambert 2005). As vigilance is higher the closer to a disturbance event (Campos and Fedigan 2014; Gaynor and Cords 2012), the observed increase in group size with time after transect cutting was likely due to guenons initially hiding in the canopy, with more individuals becoming visible with time to disturbance. Our study did not include behavioral observations, nor information about sex and age classes of the observed primates, which would have allowed investigating within-group differences in detection probability. For example, juvenile brown capuchins (*Cebus apella*) were reported feeding more often in suboptimal, risky locations, while showing lower vigilance levels (van Schaik and van Noordwijk 1989). In vervet monkeys (*Chlorocebus pygerythrus*), juveniles were the slowest age class in fleeing humans (Mikula et al. 2018). Therefore, in the guenons of our study, juveniles may have been the first to be observed after disturbance, while adult females and males were still hiding.

Average group size is estimated accurately only by independent studies involving direct follows of focal groups (Buckland et al., 2010a). However, primate group size is known to be driven by both predation risk (Croes et al. 2007; Stanford 1995) and food availability (Janson and Goldsmith 1995; Wrangham et al. 1993), which are geographically variable even within a single study area. For example, *C. ascanius* groups in Kibale, Uganda, were highly variable in number, ranging between 38 and 175 individuals (Table 3). As predation risk and food availability were likely to be geographically variable also in our study area (17,127 km^2^), we would have required following groups of different species in different areas in order to fully grasp variability in group size, which was impossible with the available resources. For this reason, we estimated group size during the survey. When comparing our observed and estimated group size (Table 2), we found that exact counts (i.e., observed group size) were consistently lower than estimates including acoustic detections, suggesting that rainforest habitat is particularly unsuitable in visually counting individual primates (Spaan et al., 2017). As it is difficult to accurately estimate the number of individuals from acoustic cues only, we suggest improving accuracy by combining visual and acoustic observations. Although our estimated group sizes were comparable to those from other sites obtained from focal groups follows (Table 3), we are aware that between–site comparisons are difficult. Depending on the composition of specific primate communities, ecological niches may or may not be occupied, affecting average group size. For this reason, in Table 3 we did not compare group sizes across allopatric species such as *L. albigena*, *P. tephrosceles*, and *Colobus guereza*, but only compared studies of the same species.

### Density estimates (D)

Considering all species, our density estimates revealed the highest density in P4 and the lowest in P2 (Table 2). When using estimated group size, ER were similar between P3 and P4, but estimates were 12% higher for the second. This substantial difference must be ascribed to larger groups observed in P4 (Fig. 3), with more individuals being spotted the more time had passed after disturbance.

At the species level, sample size in P4 was too low for three species (*P. tholloni*, *C. angolensis*, and *C. wolfi*) not allowing to model the detection probability with distance. Consequently, we could not obtain a density estimate for these species in P4. Sample size was also low (< 30 sightings; Plumptre et al. 2013) for *L. aterrimus* (*n* = 17) and *C. ascanius* (n = 18), but sufficient to fit a detection function, which however, resulted in large confidence intervals (CI) of estimated densities. As a result, the CI obtained in P3 and P4 overlapped in both species, and the resulting estimates could not be considered reliable (Plumptre et al., 2013). However, the estimated mean density was similar between passages for *L. aterrimus*, and 17% higher in P4 for *C. ascanius* (Fig. 2), consistent with the pooled analysis of all species. These results suggest an interval of 10 days on average after transect cutting (i.e., the average interval between P1 and P4) being adequate for these species. We expect the same to be true for the other species in this study, showing comparable responses to disturbance (Fig. 2 and 3). Finally, the corrected density estimates used here, were similar to those obtained in other sites from follows of focal groups (Table 3) and supported by adequate sample size (Supporting Table 2), with the sole exception of the *C. angolensis* (*n* = 23). By that they support the efficiency of LTDS as an effective tool for estimating primate density, provided adherence to the method’s best practice guidelines (Buckland et al., 2010a).

### Conclusions and practical recommendations

As expected, our study showed the negative effects of disturbance on both primate encounter rates and observed group sizes. It also showed that 24 h after disturbance were sufficient to detect most of the groups present along the transect, suggesting that primate groups should be counted not earlier than 24 h after the transect cutting. The same interval seemed adequate to assess group size of the two Colobinae, given their cohesiveness. However, due to increased vigilance after the transect cutting and / or avoidance of the area where the primates were disturbed, a longer time was required for the Cercopithecinae to resume their behavior under undisturbed conditions, allowing more accurate estimates of group size. For these species, we recommend the actual survey to take place a minimum of 7, ideally 10 days after cutting the transect, unless average group size has been quantified in a separate study involving direct follows of focal groups.

Despite efforts to minimize disturbance in our study, exact counts of group size were consistently lower than group size estimates. In situations where it is impossible or unfeasible to measure distances to individual primates, we recommend group size to be estimated combining visual and acoustic observations. To reduce bias, a team of ideally 3–5 trained observers (four in our study) should provide independent estimates of group size. As larger teams exert higher disturbance, it is important that the trade-off between number of independent estimates and disturbance is carefully weighted when designing the study. For example, a single observer could survey a transect almost unnoticed, maximizing the time spent in assessing group size, although this can be impractical for very large group sizes. Finally, estimates’ accuracy could be improved by using spatial hierarchical models, which estimate primate density by modelling detection probability and group size as a function of covariates (Cavada et al. 2017b).

By affecting both group encounter rate and group size, our study showed that observer disturbance is a critical factor to be considered in order to obtain reliable estimates of primate population density. Importantly, it also showed inter-specific differences in the way primates respond to disturbance, possibly resulting from different anti-predatory strategies as a response to hunting pressure. Some species responded by producing obvious alarm calls and subsequent hiding (e.g., *L. aterrimus*). Others, by remaining silent and gathering rather than fleeing (e.g., *P. tholloni*).

We wish to emphasize that our findings may apply differently to other areas of primate occurrence. The primates inhabiting the block South of SNP have been heavily poached in the past and still are hunted today. However, due to the remoteness and the size of the area, hunting pressure from humans was never continuous and did not affect the entire population simultaneously. In addition, only two surveys were conducted in the area before this study (Blake 2005; Grossmann et al. 2008). As a result, the groups encountered in our study, were very responsive to the presence of researchers, emitting frequent vocalizations (e.g., *L. aterrimus*) and avoiding the transect area for more than a week (e.g., all Cercopithecinae) after the first encounter with humans in P1. However, different primate communities are expected to show different behaviors according to habitat, niche occupation, and hunting pressure. Similarly, as thicker vegetation requires more cutting, the ideal interval between transect cutting and the survey will depend on the ground vegetation of the specific study area. It will also depend on the degree of habituation of the primates in study. For example, if the monkeys are used to encounter researchers (e.g., when pre-existing transects are used for monitoring purposes) their response might be reduced or not existent. It is therefore the responsibility of any future study to consider these factors when designing a primate survey using LTDS, in order to grant accurate assessments of primate density and abundance contributing to the implementation of effective conservation measures.

## Supplementary Information

Below is the link to the electronic supplementary material.

Supplementary file1 (DOCX 51 KB)

Supplementary file2 (DOCX 17 KB)

Supplementary file3 (DOCX 262 KB)

## Data Availability

The raw data used in this study are available via the Edmond Data Repository 10.17617/3.C0HBLR (Bessone et al. 2022).
