# Supplementary material for: Assessing the effects of survey-inherent disturbance on primate detectability: Recommendations for line transect distance sampling
Source: Primates. 2022 Dec 9;64(1):107–21. doi: 10.1007/s10329-022-01039-4 (PMC9842571; doi:10.1007/s10329-022-01039-4)
Supplement: Supplementary file 1 — Supplementary file1 (DOCX 51 KB) [file 10329_2022_1039_MOESM1_ESM.docx]

**Assessing the effects of survey inherent disturbance on primate detectability: recommendations for line transect distance sampling**

**Primates**

Mattia Bessone, Hjalmar S. Kühl, Gottfried Hohmann, Ilka Herbinger, K. Paul N’Goran, Papy Asanzi, Pedro B. Da Costa, Violette Dérozier, Ernest Fotsing D.B., Ikembelo Beka B., Mpongo Iyomi D., Iyomi Iyatshi B., Pierre Kafando, Mbangi Kambere A., Dissondet Moundzoho B., Musubaho Wanzalire L.K., Barbara Fruth

**Corresponding author:**  Mattia Bessone, Max Planck Institute of Animal Behavior, Department of Ecology of Animal Societies, Bücklestraße 5, 78467, Konstanz, Germany. Email _ [mbessone@ab.mpg.de](mailto:mbessone@ab.mpg.de) ; ORCID _ 0000-0002-8066-6413

**Supporting Fig 2 Differences in estimated group size between passages and species.** Left: average group size observed in each passage (P2 = orange; P3 = yellow; P4 = green) by species (row 2 to 6) and considering all species together (top row). Right: posterior distribution of pairwise contrasts of average observed group size between passage for each species considered.**
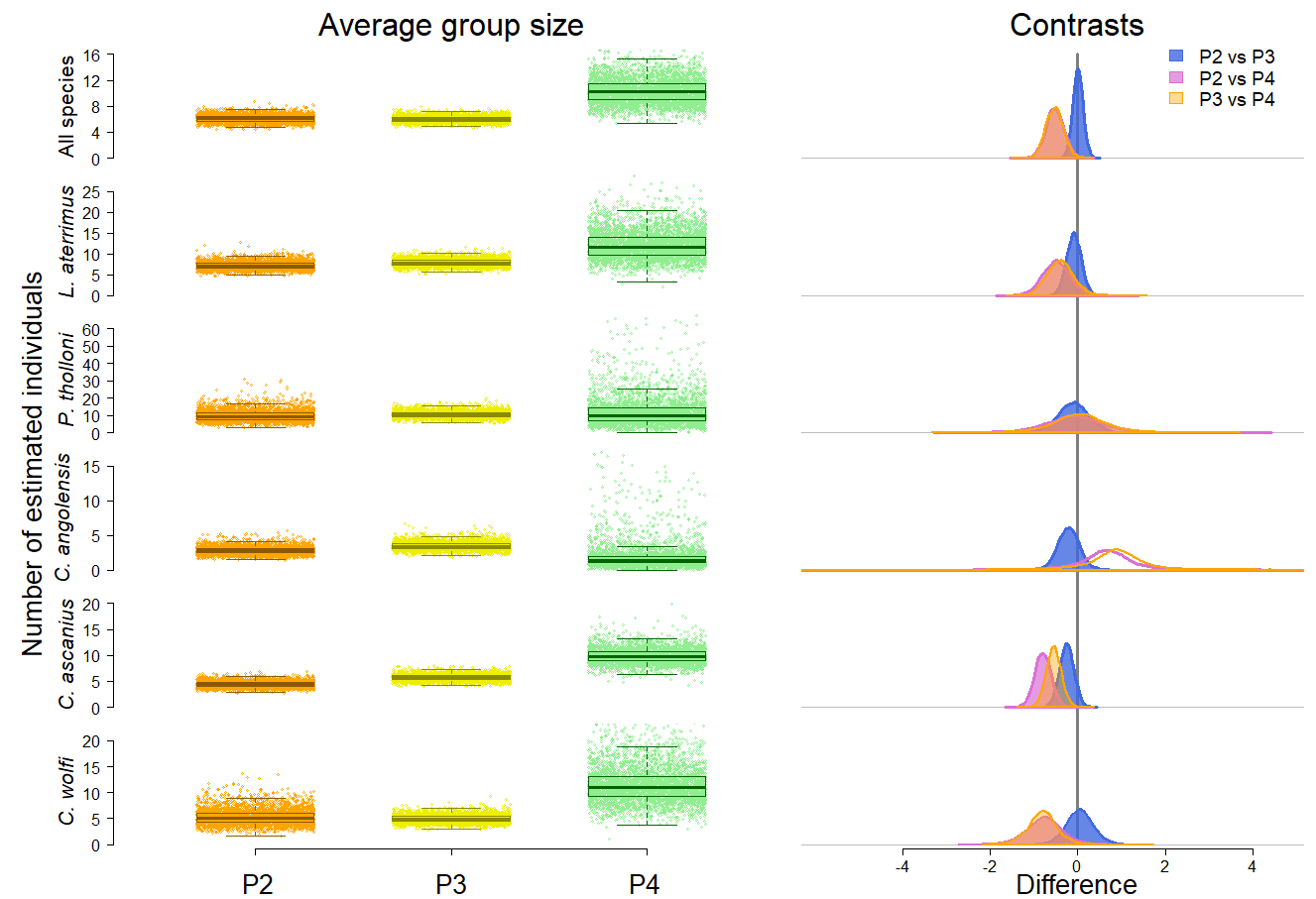
**
