# Supplementary material for: Assessing the effects of survey-inherent disturbance on primate detectability: Recommendations for line transect distance sampling
Source: Primates. 2022 Dec 9;64(1):107–21. doi: 10.1007/s10329-022-01039-4 (PMC9842571; doi:10.1007/s10329-022-01039-4)
Supplement: Supplementary file 2 — Supplementary file2 (DOCX 17 KB) [file 10329_2022_1039_MOESM2_ESM.docx]

| **Species** | **Passage** | **Groups observed** | **Vocalizations** | **Observations** | **Proportion vocalizations** |
| --- | --- | --- | --- | --- | --- |
| ***L. aterrimus*** | P1 | 204 | 156 | 48 | 0.76 |
|  | P2 | 134 | 58 | 76 | 0.43 |
|  | P3 | 199 | 109 | 90 | 0.55 |
|  | P4 | 25 | 8 | 17 | 0.32 |
| ***P. tholloni*** | P1 | 28 | 10 | 18 | 0.36 |
|  | P2 | 27 | 1 | 26 | 0.04 |
|  | P3 | 49 | 9 | 40 | 0.18 |
|  | P4 | 8 | 2 | 6 | 0.25 |
| ***C. angolenis*** | P1 | 18 | 11 | 7 | 0.61 |
|  | P2 | 17 | 2 | 15 | 0.12 |
|  | P3 | 33 | 8 | 25 | 0.24 |
|  | P4 | 2 | 0 | 2 | 0.00 |
| ***C. ascanius*** | P1 | 58 | 18 | 40 | 0.31 |
|  | P2 | 86 | 17 | 69 | 0.20 |
|  | P3 | 125 | 15 | 110 | 0.12 |
|  | P4 | 20 | 2 | 18 | 0.10 |
| ***C. wolfi*** | P1 | 56 | 34 | 22 | 0.61 |
|  | P2 | 84 | 28 | 19 | 0.42 |
|  | P3 | 47 | 35 | 49 | 0.60 |
|  | P4 | 12 | 5 | 7 | 0.42 |

**Assessing the effects of survey inherent disturbance on primate detectability: recommendations for line transect distance sampling**

**Primates**

Mattia Bessone, Hjalmar S. Kühl, Gottfried Hohmann, Ilka Herbinger, K. Paul N’Goran, Papy Asanzi, Pedro B. Da Costa, Violette Dérozier, Ernest Fotsing D.B., Ikembelo Beka B., Mpongo Iyomi D., Iyomi Iyatshi B., Pierre Kafando, Mbangi Kambere A., Dissondet Moundzoho B., Musubaho Wanzalire L.K., Barbara Fruth

**Corresponding author:**  Mattia Bessone, Max Planck Institute of Animal Behavior, Department of Ecology of Animal Societies, Bücklestraße 5, 78467, Konstanz, Germany. Email _ [mbessone@ab.mpg.de](mailto:mbessone@ab.mpg.de) ; ORCID _ 0000-0002-8066-6413

**Supporting table 1. Proportion of acoustic (heard) groups for each species and passage.** *Groups observed*: total number of group observed or heard per passage; *Vocalizations*: number of heard groups per passage; *Observations*: number of directly observed groups per passage; *Proportion vocalizations*: proportion of acoustic (heard) groups of all groups detected for each species and passage.
