# Supplementary material for: Assessing the effects of survey-inherent disturbance on primate detectability: Recommendations for line transect distance sampling
Source: Primates. 2022 Dec 9;64(1):107–21. doi: 10.1007/s10329-022-01039-4 (PMC9842571; doi:10.1007/s10329-022-01039-4)

**Assessing the effects of survey inherent disturbance on primate detectability: recommendations for line transect distance sampling**

**Primates**

Mattia Bessone, Hjalmar S. Kühl, Gottfried Hohmann, Ilka Herbinger, K. Paul N’Goran, Papy Asanzi, Pedro B. Da Costa, Violette Dérozier, Ernest Fotsing D.B., Ikembelo Beka B., Mpongo Iyomi D., Iyomi Iyatshi B., Pierre Kafando, Mbangi Kambere A., Dissondet Moundzoho B., Musubaho Wanzalire L.K., Barbara Fruth

**Corresponding author:**  Mattia Bessone, Max Planck Institute of Animal Behavior, Department of Ecology of Animal Societies, Bücklestraße 5, 78467, Konstanz, Germany. Email _ [mbessone@ab.mpg.de](mailto:mbessone@ab.mpg.de) ; ORCID _ 0000-0002-8066-6413

**Supporting table 2 Species and passage specific distance sampling models and fitted detection functions of the corrected estimates including observations from P3 and P4 (bold rows; see “Material and Methods” in main text).** For each species and passage: *Passage (observations)*: total number of observations used after truncation. *Model (a;b;c)*: selected model for density estimation showing (a) key function (HN = Half normal; HR = Hazard rate; UNI = Uniform), (b) series expansion (Cos = cosine; SP = simple polynomial). (c) adjustment terms. *Detection function of corrected estimates*: detection probability function fitted for the corrected density estimation.


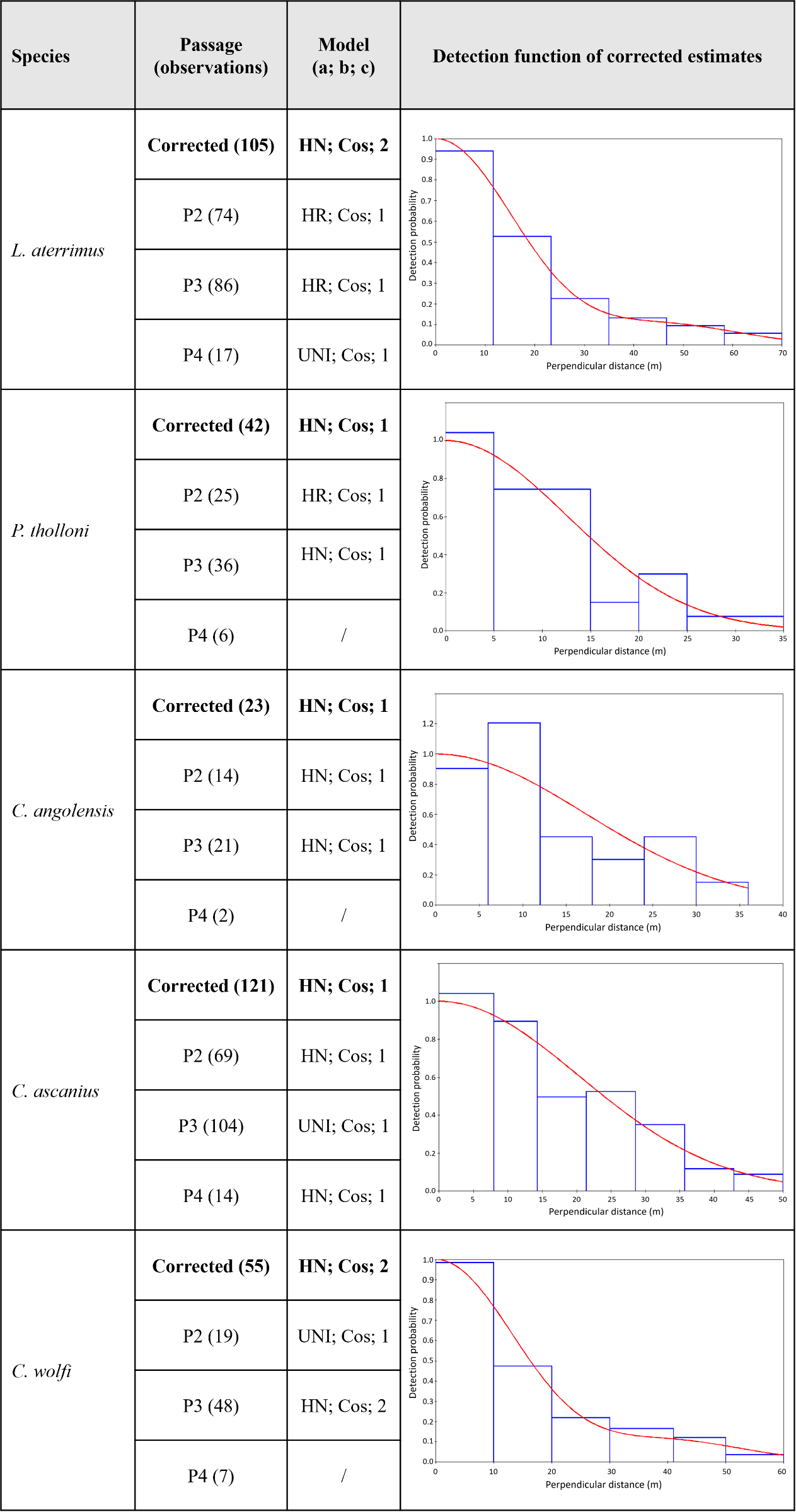

Supplement: Supplementary file 3 — Supplementary file3 (DOCX 262 KB) [file 10329_2022_1039_MOESM3_ESM.docx]
